# Numerical Analysis of Mask-Based Phase Reconstruction in Phaseless Spherical Near-Field Antenna Measurements

**DOI:** 10.3390/s25185637

**Published:** 2025-09-10

**Authors:** Adrien A. Guth, Sakirudeen Abdulsalaam, Holger Rauhut, Dirk Heberling

**Affiliations:** 1 Institute of High Frequency Technology, RWTH Aachen University, 52074 Aachen, Germany; guth@ihf.rwth-aachen.de; 2 Department of Mathematics, Ludwig-Maximilians-Universität München, 80333 München, Germany; abdulsalaam@math.lmu.de (S.A.); rauhut@math.lmu.de (H.R.); 3 Munich Center for Machine Learning, 80539 München, Germany; 4 Fraunhofer Institute for High Frequency Physics and Radar Techniques, 53343 Wachtberg, Germany

**Keywords:** antenna measurements, phaseless spherical near-field measurements, spherical near-field–far-field transformation, spherical wave expansion, phase recovery

## Abstract

Phase-retrieval problems are employed to tackle the challenge of recovering a complex signal from amplitude-only data. In phaseless spherical near-field antenna measurements, the task is to recover the complex coefficients describing the radiation behavior of the antenna under test (AUT) from amplitude near-field measurements. The coefficients refer, for example, to equivalent currents or spherical modes, and from these, the AUT’s far-field characteristic, which is usually of interest, can be obtained. In this article, the concept of a mask-based phase recovery is applied to spherical near-field antenna measurements. First, the theory of the mask approach is described with its mathematical definition. Then, several mask types based on random distributions, ϕ-rotations, or probes are introduced and discussed. Finally, the performances of the different masks are evaluated based on simulations with multiple AUTs and with Wirtinger flow as a phase-retrieval algorithm. The simulation results show that the mask approach can improve the reconstruction error depending on the number of masks, oversampling, and the type of mask.

## 1. Introduction

Spherical near-field (SNF) measurement [1] is one of the most recognized and accurate [2] techniques for evaluating the commonly desired far-field (FF) characteristics of an antenna under test (AUT). In contrast to FF techniques, near-field (NF) methods necessitate an additional post-processing step. A mathematical transformation is used to acquire the FF properties from the NF measurements. For spherical measurements, a transformation with spherical waves is often utilized. This transformation, based on the spherical wave expansion (SWE), decomposes the measured field in spherical waves so that the AUT’s radiation behavior is uniquely defined through weights of these spherical waves. However, to compute these weights, also known as spherical mode coefficients (SMCs), it is necessary to measure both amplitude and phase. This requirement is common for other transformations, such as the one based on equivalent currents.

From a measurement perspective, assessing the phase requires costly equipment and obtaining good accuracy at higher frequencies becomes a real challenge. Furthermore, in specific scenarios, such as for over-the-air measurements, no reference phase is available. Therefore, research in phaseless solutions has been of high interest for many years. A well-known approach is indirect holography, which can be implemented either with a reference antenna [3] or a synthetic reference wave [4]. However, these approaches require non-overlapping spectra of the reference antenna and AUT. This method can be improved by using a synthetic reference wave based on phase shifters. Moreover, a novel variant exists, but only for broadband antennas [5], where phase retrieval does not occur in the plane wave spectrum but in the time domain. Other approaches are mainly based on an iterative phase-retrieval algorithm with additional measurements. Different algorithms are used, such as PhaseLift [6], Wirtinger flow [7], or a variant involving Gerchberg–Saxton [8,9]. The first is a convex algorithm, lifting the original problem in higher dimension. The second is a non-convex algorithm which suffers from becoming trapped in local minima. In [10], the performances of Gerchberg–Saxton, Fienup, reweighted amplitude flow, and reweighted Wirtinger flow applied to planar NF measurements are compared in the dependency of initialization methods and measurement parameters. Besides the use of an iterative phase-retrieval algorithm, additional measurements are introduced in all applications to enhance the amount of information and the number of independent measurements. The most popular procedure is the two-scan method, for which measurements are taken either on two spheres [9,11], two planes [10], a sphere and a plane [12], or a sphere and a polyhedron [13]. In [14], multiple probes are used; in [15,16], multiple AUT positions were used. Moreover, the multiple probe approach was also used to establish partially coherent phase relations [17,18]. In this way, the phase-retrieval problem can be linearized at the cost of using a multiple-channel receiver. Coherent phase relations can also result from multi-frequency measurements [19].

In a context not specific to antenna measurements, the authors in [20] introduced a framework combining PhaseLift and multiple structured illuminations. The measurement setup consists of a source that illuminates an object from which the pattern is measured. Structured illuminations can be generated, for example, through masking, optical grating, ptychography, or oblique illuminations, and they have found applications in fields such as X-ray crystallography and optics. Furthermore, in antenna measurements, the previously mentioned methods, such as two-scan or multiple probes, can be interpreted as structured illuminations. In [21], the structured illuminations are generalized to the concept of coded diffraction patterns (CDPs) described through a problem formulation, resulting in the CDPs being a modulation of the spectrum to be recovered. The authors have demonstrated that, under the application of CDPs and PhaseLift, the resulting convex relaxation is exact.

In our work, the concept and mathematical formulation of CDPs is applied to SNF antenna measurements and its NF–FF transformation. The transformation used is based on the SWE [1]. Numerical experiments on random and probe-based masks were performed in [22] and [23], respectively. These investigations are continued and extended in this work with new AUTs and deeper parameter analysis. Wirtinger flow, an algorithm for non-convex phase-retrieval problems, is used for the experiments, since it offers a faster solution than PhaseLift.

## 2. Phaseless Spherical Near-Field Measurements

In SNF antenna measurements, several methods exist to compute the FF radiation characteristic of an AUT from NF measurements. One of these methods consists of describing the AUT’s radiation behavior using spherical waves. Therefore, each AUT can be represented by weights of spherical waves. The relationship between the radiated field measured by the probe antenna *w* and the weights $T_{smn}$, also called SMCs, is given by the transmission formula in [1], defined as

(1) $$w\left(A,\chi ,\theta ,\phi \right)=v\sum_{s=1}^{2}\sum_{n=1}^{N}\sum_{m=-n}^{n}T_{smn}e^{im\phi }\sum_{\mu =-\nu_{\max}}^{\nu_{\max}}d_{\mu m}^{n}\left(\theta \right)e^{i\mu \chi }P_{s\mu n}\left(kA\right).$$

In the equation above, (A,χ,θ,ϕ) are the measurement coordinates and polarization, $e^{im\phi }d_{\mu m}^{n}\left(\theta \right)e^{i\mu \chi }$ are the Euler rotations, and $P_{s\mu n}\left(kA\right)$ are the probe response constants (PRCs). The PRCs represent the probe effect in the measurements through

(2) $$P_{s\mu n}\left(kA\right)=\frac{1}{2}\sum_{\sigma =1}^{2}\sum_{\nu =|\mu |}^{\nu_{\max}}C_{\sigma \mu \nu }^{sn(3)}\left(kA\right)R_{\sigma \mu \nu },$$

where $C_{\sigma \nu \mu }^{sn(3)}\left(kA\right)$ is a translation coefficient and $R_{\sigma \mu \nu }$ corresponds to the probe SMCs in receiving mode. A compact form of (1) is found by combining the sum indices smn to *j* so that $\sum_{s=1}^{2}\sum_{n=1}^{N}\sum_{m=-n}^{n}=\sum_{j=1}^{J}$ resulting in a matrix equation

(3) w=Ψq,

with $w={\left[w\left(A_{1},\chi_{1},\theta_{1},\phi_{1}\right),\ldots ,w\left(A_{M},\chi_{M},\theta_{M},\phi_{M}\right)\right]}^{T}\in C^{M}$, $\Psi \in C^{M\times J}$ and $q={\left[vT_{1},\ldots ,vT_{J}\right]}^{T}\in C^{J}$.

In the case where phaseless (amplitude-only) measurements are available, (3) can be rewritten as

(4) $$\left|w\right|=\left|\Psi q\right|.$$

The aim is to reconstruct the complex SMCs q from amplitude near-field measurements $\left|w\right|$.

## 3. Masks

In [21], CDPs were introduced as the outcome of a modulating wave form of the signal to be recovered and are described as follows:

(5) $$y_{m,l}={\left|f_{m}^{*}D_{l}^{*}x\right|}^{2}.$$

$y_{m,l}$ is the measurement *m* with mask *l*, $D_{l}$ is a diagonal matrix with the modulation pattern, and $f_{m}^{*}$ is a row of the transformation matrix. The discrete Fourier transform is used as the transformation. The square in (5) is due to the measured quantity, which is intensity. Now, considering the problem definition (4) for a single measurement $w_{m,l}$ and introducing the modulating wave form results in

(6) $$\left|w_{m,l}\right|=\left|\Psi_{m}H_{l}q\right|,$$

where $\Psi_{m}$ is a row of Ψ. In this work, the modulating wave form is referred to as a mask characterized by $H_{l}\in C^{J\times J}=\mathrm{diag}\left(h_{1},h_{2},\ldots ,h_{J}\right)$. With w containing multiple measurements, (6) corresponds to

(7) $$\left|w_{l}\right|=\left|\Psi H_{l}q\right|.$$

Typically, to improve the reconstruction performance, several measurements with different masks are combined so that (7) is extended to

(8) $$\left|\tilde{w}\right|=\left|{\left[\Psi H_{l}\right]}_{l=1}^{L}q\right|,$$

or compactly,

(9) $$\left|\tilde{w}\right|=\left|\mathrm{Aq}\right|,$$

where $\tilde{w}={\left[w_{1},\ldots ,w_{L}\right]}^{T}\in C^{ML}$ and $A={\left[\Psi H_{1},\ldots ,\Psi H_{L}\right]}^{T}\in C^{ML\times J}$. *L* denotes the number of masks and $M_{\mathrm{total}}=ML$ is the total number of measurements. From a practical standpoint, each mask defines a measurement scenario in which a set of measurements, usually following a fixed sampling grid, is taken. All sets of measurements $w_{l}$ are then stacked up in $\tilde{w}$. From a transformation perspective, each single set $w_{l}$ corresponds to the combination of a transformation matrix Ψ and a specific mask $H_{l}$. Since the sampling between measurement scenarios remains the same, Ψ is not changing and $H_{l}$ is almost surely unique.

In general, the phase-retrieval problem as described in (4) is strongly dependent on two factors: the number of measurements and the number of unknowns. A challenge is then to find the sample complexity, which is for a fixed number of unknowns, the number of measurements that is sufficient to achieve a successful recovery. It is similar to the mask approach, with the difference that the total number of measurements is linked to the number of measurements per mask and the number of masks. The existing theory provides a sample complexity for the discrete Fourier transform [20,21,24,25] in combination with PhaseLift. However, to the best of our knowledge, for the SWE, no sample complexity is available in the literature. Although, it is expected that it will not be the same.

The following section introduces several types of masks. Firstly, masks based on random distribution are shown. They demonstrate the applicability of CDPs to SNF antenna measurement. The results of random masks are used to benchmark new mask approaches. Secondly, masks from ϕ-rotations are discussed. Lastly, probe-based masks are presented, describing a more practical approach to implementing masks.

### 3.1. Random Masks

Two types of random masks with different distributions are investigated.

#### 3.1.1. Gaussian and Uniform Masks

In our first analysis, Gaussian masks, with entries $h_{j}$, following standard complex normal distributions and are defined as

(10) $$h_{j}\in N_{C}\left(0,1\right),$$

are evaluated. In addition, masks based on a uniformly distributed phase (11), amplitude (12), phase and amplitude (13) as well as on a Gaussian-distributed amplitude (14) described through

(11) $$\begin{array}{cc} h_{j}=e^{ix_{j}} & \;\mathrm{with}\;x_{j}\in U\left(0,2\pi \right),\;\;\;\;\;\;\;\; \end{array}$$

(12) $$\begin{array}{cc} h_{j}\;=\;x_{j} & \;\mathrm{with}\;x_{j}\in U\left(0,1\right),\;\;\;\;\;\;\;\;\; \end{array}$$

(13) $$\begin{array}{cc} h_{j}\;=\;x_{j}e^{iy_{j}} & \mathrm{with}\;x_{j}\in U\left(0,1\right)\;\mathrm{and}\;y_{j}\in U\left(0,2\pi \right), \end{array}$$

(14) $$\begin{array}{cc} h_{j}\;=\;x_{j} & \;\mathrm{with}\;x_{j}\in N\left(0,1\right),\;\;\;\;\;\;\;\;\; \end{array}$$

are also analyzed.

#### 3.1.2. Categorical Masks

Next, a similar formulation is considered, whereby the mask entries $h_{j}$ follow a categorical distribution. So, each $H_{l}$ is drawn from $H=\sum_{j=1}^{J}h_{j}e_{j}e_{j}^{*}$, where ${\left\{h_{j}\right\}}_{j=1}^{J}$ are independent copies of a complex random variable *h* which obeys

(15) $$\begin{array}{c} E[h]\;=\;E[h^{2}]=0,\\ |h|\;\leq \;b\;\mathrm{almost}\;\mathrm{surely}\;\mathrm{for}\;\mathrm{some}\;b>0,\\ E[h^{4}]=2E{\left[h^{2}\right]}^{2}\;\mathrm{and}\;\nu :=E\left[h^{2}\right]. \end{array}$$

An example of a complex random variable *h* which satisfies (15) can be

(16) $$h=\left\{\begin{array}{c} 1,\;\;\frac{1}{8},\\ -1,\;\frac{1}{8},\\ 0,\;\;\frac{1}{2},\\ i,\;\;\frac{1}{8},\\ -i,\;\frac{1}{8}. \end{array}\right.$$

The above distribution has been introduced in [24]. Thus, in this case, each modulation is given by a categorical random vector. The resulting distribution from (16) will be used in our investigations in Section 4.

### 3.2. ϕ-Rotations Masks

Now, we take a closer look at the SWE (1) and its matrix form (3). The term containing the ϕ dependency is $e^{im\phi }$. Besides ϕ, this term is only dependent on the index *m*. Therefore, it can be extracted from Ψ, so that

(17) $$w=\Psi_{-\phi }\mathrm{Hq}$$

holds. The entries of H are defined through $h_{j}=e^{im\phi }$ and $\Psi_{-\phi }$ represents the basis without ϕ dependency. We now consider H as a mask and aim to generate several different masks. Two parameters are taken into account: *m* and ϕ. Since *m* is an index and is related to *j*, its value is determined by its position in H according to the entry of q. The range is given by $m\in \left[-N,N\right]$, but its value distribution is fixed and given by its position in H, which is inappropriate to generate multiple different masks. On the other hand, ϕ is given through the current measurement. Therefore, each batch of measurements with a constant ϕ characterizes a mask. However, this kind of mask can be viewed as oversampling, which has been shown to improve the phaseless reconstruction only to a limited extent [8,9,22]. For these reasons, this mask approach has not been investigated further.

### 3.3. Probe-Based Masks

Finally, a more practical approach based on probes is considered. As introduced in [26], the PRCs can be extracted from the transformation basis so that

(18) $$w=\Psi_{-p}\left(p\circ q\right),$$

where $\Psi_{-p}$ is the basis without PRCs, $p\in C^{J}$ and ∘ represents the Hadamard product, which is an element-wise product of both vectors. (18) can be rewritten to

(19) $$w=\Psi_{-p}\mathrm{Pq},$$

with $P=\mathrm{diag}\left(p_{1},p_{2},\ldots ,p_{J}\right)\in C^{J\times J}$. One can see that P is equivalent to a mask H according to the definition in (7). However, the extraction of the PRCs is only valid for $\nu_{\max}=1$, so that only a first-order probe (FOP), which can be accurately approximated by solely taking into account μ=±1, complies.

Nevertheless, a definition of probe-based masks with high-order probe (HOP) correction is possible under certain modifications of the initial problem. From (1), it can be seen that the PRCs $P_{s\mu n}\left(kA\right)$ are dependent on μ together with $d_{\mu m}^{n}\left(\theta \right)e^{i\mu \chi }$, so that its solo extraction in (3) is not possible. However, we can redefine (3) to

(20) $$w=\Psi^{\prime }q^{\prime },$$

with $q^{\prime }\in C^{J^{\prime }}$, $\Psi^{\prime }\in C^{M\times J^{\prime }}$ and $J^{\prime }=J\left(2\nu_{\max}+1\right)$. Accordingly, the second dimension of $\Psi^{\prime }$ is now built over $\sum_{smn\mu }=\sum_{J^{\prime }}$ instead of $\sum_{smn}=\sum_{J}$ as it is the case for Ψ. The number of unknowns increased exponentially, but since $T_{smn}$ is not dependent on μ, $q^{\prime }$ is enlarged through duplicates of the entries of q.

Besides the above comparisons on the equivalence in terms of definition, two aspects need to be discussed when it comes to the application of these masks. The PRCs describe the translation of the probe to the measurement surface. Therefore, the PRCs depend on the measurement radius and the probe’s SMCs. Regarding the latter, to generate different masks, one has to build several probes with various SMCs distributions and thus, different radiation characteristics. The choice of measurement radius is also crucial, since the probe effect decreases with increasing radius.

To investigate the effectiveness of this approach, two types of masks based on random and patch array probes are introduced.

#### 3.3.1. Random Probes

Random probes are generated so that their SMCs $q^{\mathrm{probe}}$ follow a standard normal complex distribution. Thus, the SMCs entries are given through

(21) $$q_{i}^{\mathrm{probe}}\in N_{C}\left(0,1\right).$$

According to this distribution, two types of probes are built: FOP and HOP. For FOP, only elements for m=±1 are chosen according to the above distribution, whereas for HOP, all elements comply with (21).

#### 3.3.2. Patch Array Probes

The second type of probe-based masks has a higher practical relevance. Here, probes based on patch arrays are created. Arrays offer the advantage of generating antennas with diverse radiation characteristics by appropriately exciting each array element. Considering a patch antenna array consisting of $N_{a}\times N_{a}$ elements, each located at $\left(x_{n_{x}},y_{n_{y}}\right)$. The total radiated field of the array is described by

(22) $$\begin{array}{c} E_{\mathrm{tot}}\left(\theta ,\phi \right)=E_{\mathrm{el}}\left(\theta ,\phi \right)AF\left(\theta ,\phi \right), \end{array}$$

where $E_{\mathrm{el}}\left(\theta ,\phi \right)$ is the field of a single array element and $AF\left(\theta ,\phi \right)$ is the array factor. To create multiple probes and thus multiple masks, the signal fed to each element is randomly weighted according to

(23) $$\begin{array}{cc} AF\left(\theta ,\phi \right)=\sum_{n_{x}=1}^{N_{a}}\sum_{n_{y}=1}^{N_{a}} & w_{n_{x},n_{y}}e^{i\left(n_{x}\Delta \varphi_{x}+n_{y}\Delta \varphi_{y}\right)}e^{ik\left(x_{n_{x}}\sin\left(\theta \right)\cos\left(\phi \right)+y_{n_{y}}\sin\left(\theta \right)\sin\left(\phi \right)\right)}, \end{array}$$

where *k* is the propagation constant, and the weights follow the distributions

(24) $$\begin{array}{cc} \; & w_{n_{x},n_{y}}=x+iy\;\mathrm{with}\;x,y\in U\left(0,1\right), \end{array}$$

(25) $$\begin{array}{cc} \; & \Delta \varphi_{x},\Delta \varphi_{y}\in U\left(0,2\pi \right). \end{array}$$

The random factor from (24) affects the side lobe distribution and intensity, while the ones of (25) impact the beam tilt in *x* and *y* direction.

In the next section, numerical experiments will be conducted to evaluate the performance of the previously introduced mask types.

## 4. Numerical Results

**Figure 1:**
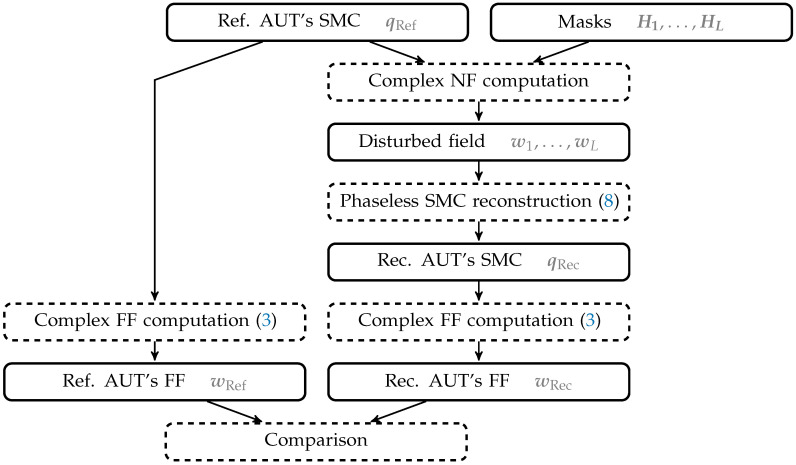
Simulation setup.

In this section, the reconstruction performance of the masks previously introduced and defined is evaluated based on numerical experiments. To achieve this, the simulation setup illustrated in Figure 1 is used. AUTs with known SMCs from measurements are taken, and masks are generated. Subsequently, both are combined to compute a disturbed NF, i.e., one affected by the mask, on a given sampling scheme. Afterwards, the NF phase is omitted and a phase-retrieval algorithm is applied to reconstruct the original complex SMCs. Finally, the FF is computed from the resulting SMCs and compared to the FF obtained with the original SMCs according to a specific metric.

**Figure 2:**
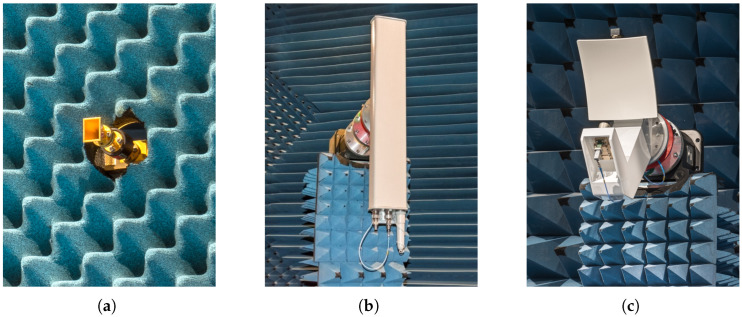
AUTs used in investigations: (**a**) SGH, (**b**) BS, and (**c**) mmVAST.

During the experiments, a standard gain horn (SGH), a basis station (BS), and a reflector antenna (mmVAST) are used. The AUTs are shown in Figure 2 and their main parameters and characteristics are listed in Table 1. The diversity in the AUTs through different directivities and beam tilts emphasize the limits or robustness of the mask approach. The SGH and mmVAST, with a rotational symmetry in their radiation pattern and high–very high directivity, are expected to be good candidates for phaseless characterization. In contrast, the BS with a beam tilt of $\theta =12^{\circ }$ is a more critical use case [8].

The reconstruction performance of the masks are analyzed mainly depending on two parameters. The main questions to be answered concerning the mask approach are as follows: Which type of mask is effective in achieving good FF reconstruction? How many masks are necessary to deliver a good FF reconstruction? Therefore, the first parameter is number of masks, *L*. The second one, which is important in general when it comes to phaseless characterization [8], is the number of measurements, *M*. Thus, for a given measurement scenario and mask type, both parameters will be varied and the reconstruction errors are compared. The number of measurement points will be described through an oversampling factor, δ, which is related to the number of unknowns, i.e., SMCs, *J*. Moreover, throughout the experiments, for a given oversampling, the number of measurements is split equally among the single mask measurements to enable a fair comparison of the reconstruction error with regard to the number of masks. Consequently, the number of measurements per mask is defined through $M_{l}=2\left\lceil \frac{J}{2}\frac{\delta }{L}\right\rceil$. The ceiling, and factor 2 and 1/2 are necessary since two polarizations $\chi =\left[0,\pi /2\right]$ are measured at each coordinate. The number of coordinates is thus $M_{l}/2$ and needs to be an integer. The total number of measurements is hence $M=\sum_{l=1}^{L}M_{l}=LM_{1}$. The measurement coordinates are distributed on a sphere following a spiral distribution. A spiral sampling scheme, as shown in [22,27], has a better reconstruction performance than schemes such as equi-angular or random. Since random distributions are involved, for each parameter combination, $\left(L,\delta \right)$, 10 simulations are performed with different masks. Fewer simulations per parameter combinations were considered only in the case of mmVAST, due to higher computational resources requirement. The number of different simulations is probably not statistically significant enough, but it shows preliminary results.

The phase retrieval is performed using Wirtinger flow [7]. The implementation in the PhasePack [28] with respective step-size policy and a weighted spectral initialization is used. A maximum of 5000 iterations has shown to be a good compromise between reconstruction error and computation time. A very low minimum step-size of $10^{-19}$ is chosen to enforce the number of iterations as the main stopping criterion.

Finally, the reconstructed FF is evaluated using the equivalent error signal (EES) metric defined as

(26) $$\epsilon \left(\theta_{i},\phi_{i},\chi_{i}\right)=\frac{\left|\right|w^{FF,dir,ref}\left(\theta_{i},\phi_{i},\chi_{i}\right)\left|\;-\;\right|w^{FF,dir}\left(\theta_{i},\phi_{i},\chi_{i}\right)\left|\right|}{{∥w^{FF,dir,ref}∥}_{\infty }},$$

where $w^{FF,dir,ref}\left(\theta_{i},\phi_{i},\chi_{i}\right)$ and $w^{FF,dir}\left(\theta_{i},\phi_{i},\chi_{i}\right)$ are the directivity-normalized reference and reconstructed FF, respectively. To evaluate multiple simulations with a single value metric, the mean EES

(27) $$\epsilon_{mean}=20\log_{10}\left(\frac{{∥\epsilon ∥}_{1}}{M}\right)$$

in decibel is used, where $\epsilon ={\left[\epsilon \left(\theta_{1},\phi_{1},\chi_{1}\right),\ldots ,\epsilon \left(\theta_{M},\phi_{M},\chi_{M}\right)\right]}^{T}$, while *p* simulations are performed, the resulting mean EES are merged to $\epsilon_{mean}={\left[\epsilon_{mean,1},\ldots ,\epsilon_{mean,p}\right]}^{T}$, which will be useful for subsequent analysis. An additional metric based on the SMCs and defined as

(28) $$\epsilon^{SMC}=20\log_{10}\left(\frac{∥q^{ref}{\;-\;q∥}_{2}}{∥q^{ref}{∥}_{2}}\right)$$

will also be used to assess directly the complex recovery error of the phase retrieval.

### 4.1. Random Masks

**Figure 3:**
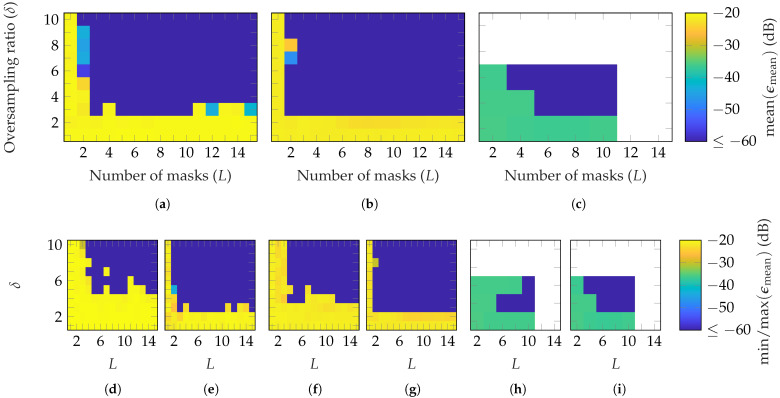
FF reconstruction error of (**a**,**d**,**e**) SGH, (**b**,**f**,**g**) BS, and (**c**,**h**,**i**) mmVAST with random masks based on a Gaussian distribution. Over 10 (SGH and BS) and 3 (mmVAST) simulations with different masks (**a**–**c**) shows the mean, (**d**,**f**,**h**), the max, and (**e**,**g**,**i**) the min of $\epsilon_{mean}$.

First, simulations are performed with random masks based on a Gaussian distribution. Figure 3 shows the reconstruction error for the three AUTs of Table 1 in dependency of the number of masks *L* and oversampling δ. Since the mmVAST has a mode truncation of N=160, much higher computational resources are necessary to process a simulation compared to the other AUTs. Therefore, only the most important parameter combinations were simulated. Moreover, the simulations were repeated with different masks only three times, instead of ten, as for the SGH and BS. The measurement radius is set to $r=0.2\;m=0.67r_{FF}$, $r=5\;m=0.42r_{FF}$ and $r=5\;m=0.07r_{FF}$ for the SGH, BS and mmVAST, respectively. In Figure 3, the mean Figure 3a–c; max Figure 3d,f,h and min Figure 3e,g,i of $\epsilon_{mean}$ are plotted for each AUT. It can be observed that on average, for the SGH, (L≥3, δ≥3) is necessary to reach an $\epsilon_{mean}\leq -60\;\mathrm{dB}$. For the BS, already, L≥2 is sufficient to obtain the same reconstruction error. Lastly, for the mmVAST, on average, (L≥4, δ≥4) lead to an EES below −60 dB, except for (L=4, δ=4). Due to the lower number of simulations performed, no detailed comparison with the SGH and BS can be made. However, generally speaking, on average, a good reconstruction with $\epsilon_{mean}\leq -60\;\mathrm{dB}$ can be achieved for all AUTs with an oversampling of δ=4 and masks between 2 and 6. From Figure 3d,f,h and Figure 3e,g,i; it can be observed that the reconstruction error is dependent on the chosen masks. Among the ten different simulations, higher oversampling and more masks are necessary for a reliable reconstruction. Furthermore, there is no guarantee that the reconstruction error will always be improved with an additional mask. The same applies to oversampling.

**Figure 4:**
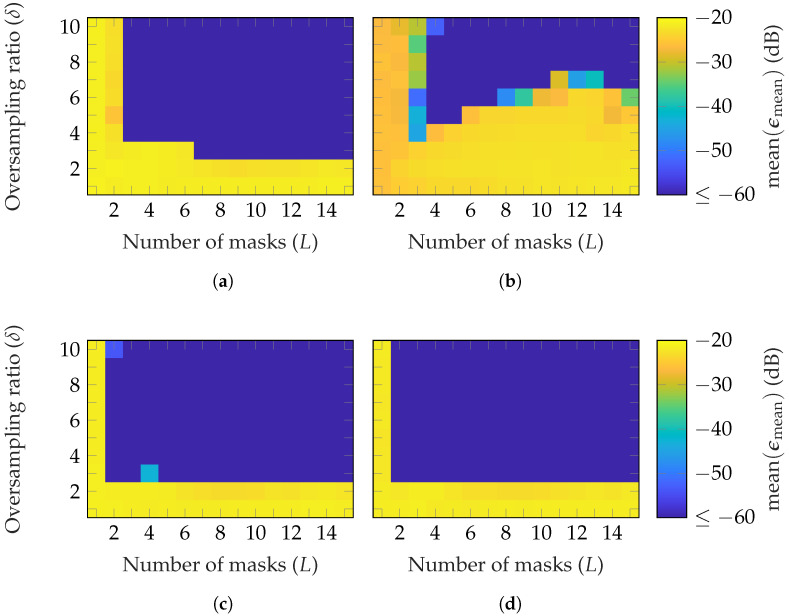
FF reconstruction error of the BS with random masks based on different distributions: (**a**) uniform phase, (**b**) uniform amplitude, (**c**) uniform phase and amplitude, and (**d**) standard normal amplitude.

In Figure 4, the reconstruction performance of different types of random masks is compared. It can be noticed that, on average, fewer masks and oversampling iterations are necessary to reach a good reconstruction with masks of uniformly distributed phases Figure 4a than amplitudes Figure 4b. However, combining both shown in Figure 4c helps to decrease the minimum required number of masks and oversampling to (L=2, δ=3). Moreover, it is worth noting that, with masks from uniformly distributed amplitudes, a higher oversampling is needed while increasing the number of masks. In Figure 4d, masks with a standard normal distribution of the amplitude are used. On average, a good reconstruction is achieved with the same parameter combinations as with uniformly distributed phase and amplitude. It should be noted that a standard normal distribution of the amplitude also includes a phase shift, since negative amplitude values result in a $180^{\circ }$ phase shift. Two potential interpretations can be made. The first one is that the standard normal distribution, compared to the uniform distribution, improves the reconstruction. The second is that a binary random phase shift, compared to a continuous phase shift, is sufficient to achieve a good reconstruction.

**Figure 5:**
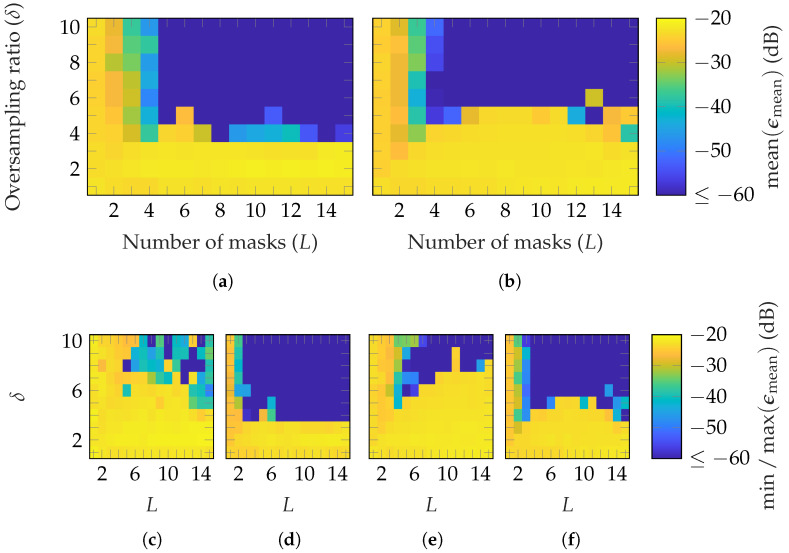
FF reconstruction error of the (**a**,**c**,**d**) SGH and (**b**,**e**,**f**) BS with random masks from a categorical distribution.

The reconstruction error of categorical distributed masks is depicted in Figure 5. On average, a higher number of masks and oversampling is needed to reach $\epsilon_{mean}\leq -60\;\mathrm{dB}$ than for Gaussian masks. This is probably due to the limited randomness through the quantization of uniform values. Furthermore, in [22], it was observed that increasing the number of masks gradually improves the reconstruction error. This behavior is not so clearly recognizable here. The transition between a bad reconstruction and a good reconstruction is more abrupt. However, it is not as abrupt as it is for Gaussian masks. A possible reason for that is the different metric and simulation setups regarding mask selection.

**Figure 6:**
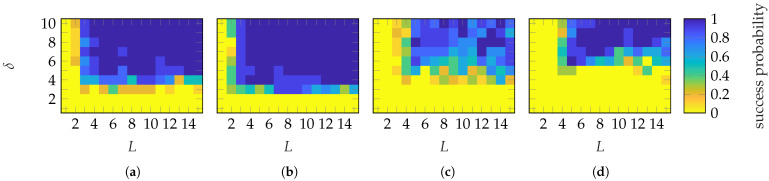
Success probability of the (**a**,**c**) SGH and (**b**,**d**) BS with (**a**,**b**) Gaussian and (**c**,**d**) categorical distributed masks.

To establish sample complexity (numerical recovery guarantee), Figure 6 shows the probability of success for each (L,δ) of the FF reconstruction for the 10 simulations. A success is defined with $\epsilon_{mean}\leq -60\;\mathrm{dB}$. An example of FF reconstructions with different $\epsilon_{mean}$ will be shown in Section 4.2. It can be observed that a successful reconstruction with a probability of 1 is achieved using Gaussian masks when (L≥5, δ≥5) and (L≥4, δ≥4) (approx.) for the SGH and BS, respectively. Categorical masks show no abrupt transition to a success probability of 1 for the SGH. For the BS, a transition is observed when (L≥5, δ≥6). However, increasing the number of masks above 6 necessitates a higher oversampling, which is generally not an expected behavior. The transition behavior of Gaussian masks is abrupt, while for categorical masks, it is mask-dependent. This is probably due to the limited level of randomness of categorical masks.

**Figure 7:**
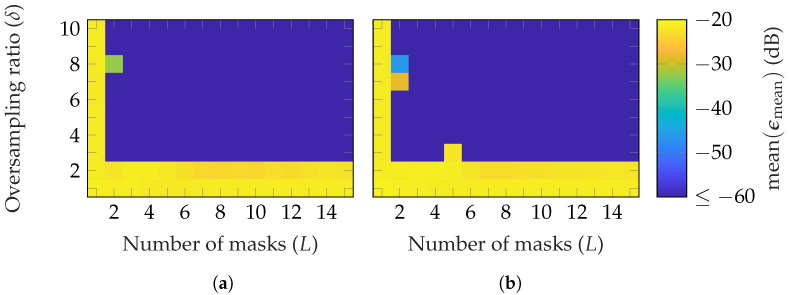
FF reconstruction error of the BS with random masks for different NF radii: (**a**) $r_{NF}=2.5\;m=21r_{FF}$ (**b**) $r_{NF}=10\;m=83r_{FF}$.

In Figure 7, the reconstruction error for different radii is depicted. As can be observed, on average, the number of masks and oversampling to achieve an $\epsilon_{mean}\leq -60\;\mathrm{dB}$ is nearly the same for all radii. This is because the effect of random masks is not radius-dependent, unlike the case for probe-based masks, which will be analyzed later.

### 4.2. Probe-Based Masks

**Figure 8:**
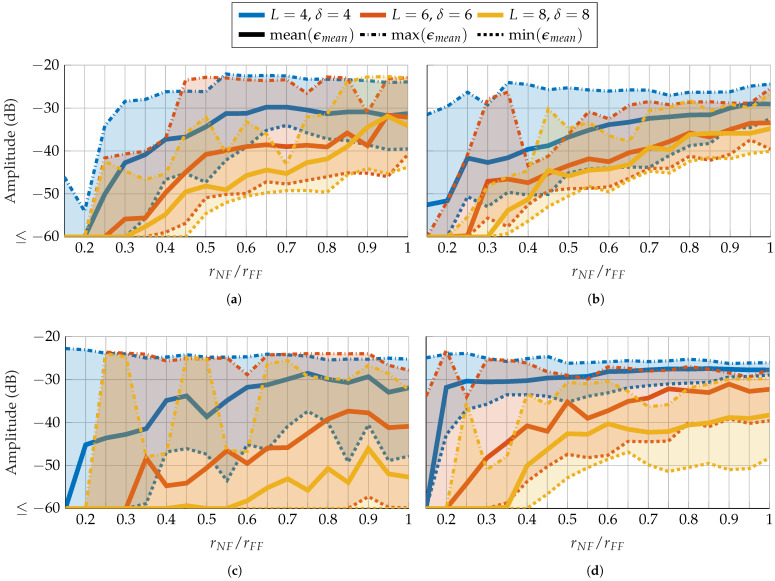
FF reconstruction error of (**a**,**c**) SGH and (**b**,**d**) BS with random probe-based masks from (**a**,**b**) FOPs and (**c**,**d**) HOPs for different combinations of number of masks and oversampling in dependency of the radius.

After demonstrating that the mask technique can improve reconstruction, a more practical approach of masks based on probes is explored. In the first part, probes based on a random distribution are treated. As mentioned in Section 3.3, the greater the distance between AUT and probe, the smaller the effect of the probe on the measured radiation pattern. Therefore, a first analysis of the radius-dependent reconstruction error is performed. For this, three parameter combinations, (L=4, δ=4), (L=6, δ=6), and (L=8, δ=8), are chosen corresponding to three different expected reconstruction errors. Figure 8 shows the error in dependence on the measurement radius. On average, the error increases as the radius increases. With FOPs, as in Figure 8a,b, the mean EES over radius of each parameter combination is more similar to one another than for the HOPs in Figure 8c,d. On average, for FOPs and HOPs as well as for SGH and BS, an $\epsilon_{mean}\leq -60\;\mathrm{dB}$ is achieved with $r_{NF}\leq 0.3r_{FF}$ for (L=8, δ=8) and $r_{NF}\leq 0.2r_{FF}$ for (L=6, δ=6). Moreover, HOPs appear to be more vulnerable to an inappropriate choice of mask since the max EES is high over a wider radius range and more fluctuating than for FOPs. It should also be noted that the used FOPs and HOPs have a mode truncation equal to that of the AUT. This is also affecting the reconstruction, as will be shown with probe-based masks from patch arrays. For further simulations, radii of $r_{NF}=0.06\;m=20$ and $r_{NF}=1.8\;m=15$ are chosen for the SGH and BS, respectively.

**Figure 9:**
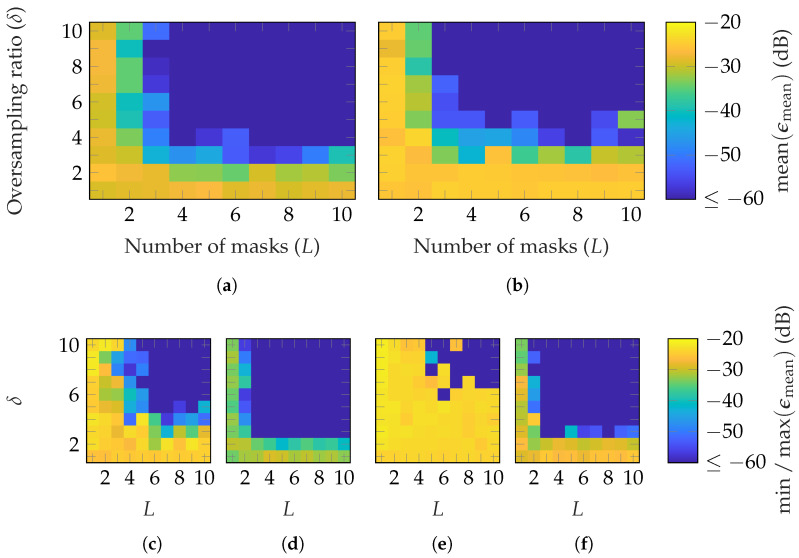
FF reconstruction error of SGH with probe-based masks from (**a**,**c**,**d**) FOPs and (**b**,**e**,**f**) HOPs with SMCs drawn from a random distribution.

**Figure 10:**
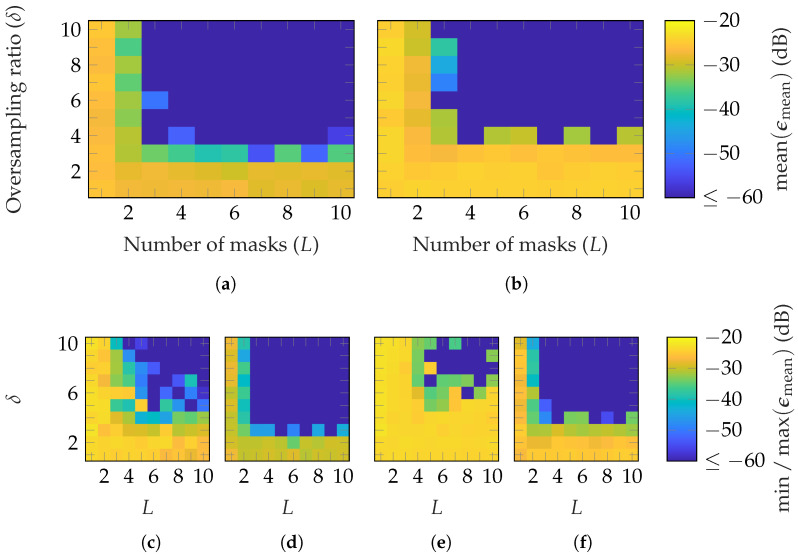
FF reconstruction error of BS with probe-based masks from (**a**,**c**,**d**) FOPs and (**b**,**e**,**f**) HOPs with SMCs drawn from a random distribution.

In Figure 9 and Figure 10, the reconstruction performance of the SGH and BS, with random probe-based masks, based on FOPs and HOPs, are shown. Similar results for both AUTs are observed, with slightly worse parameter choices for perfect reconstruction with the BS. A little fewer masks and oversampling are necessary with FOPs than HOPs to obtain a reconstruction error below −60 dB. This is valid for the average, but also for the best case: Figure 9d,f and Figure 10d,f. For the worst case, shown in Figure 9c,e and Figure 10c,e, the difference is higher. The results demonstrate that a good reconstruction is achievable with this mask approach. On average, for FOPs, (L≥4, δ≥4) and (L≥3, δ≥4) are required to reach $\epsilon_{mean}\leq -60\;\mathrm{dB}$ for the SGH and BS, respectively. Compared to random masks, it means for both AUTs, one additional mask and an increase in the oversampling factor of one are required. For the HOPs case, one more mask (for SGH) and two (for BS), along with an oversampling factor increase of two, are necessary. Moreover, it is worth noting that the transition between a bad reconstruction with $\epsilon_{mean}\geq -30\;\mathrm{dB}$ and a very good reconstruction with $\epsilon_{mean}\leq -60\;\mathrm{dB}$ is less abrupt than for random masks.

**Figure 11:**
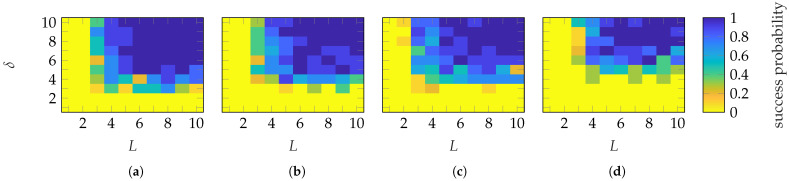
Success probability of the (**a**,**c**) SGH and (**b**,**d**) BS with (**a**,**b**) FOP and (**c**,**d**) HOP masks.

**Figure 12:**
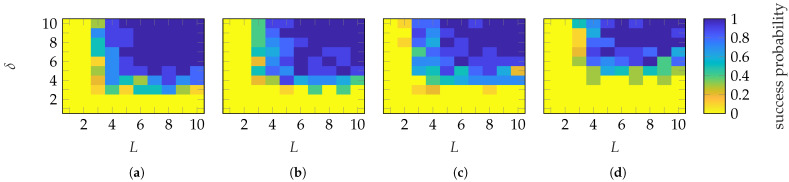
Success probability based on the SMCs metric ($\epsilon^{SMC}$) of the (**a**,**c**) SGH and (**b**,**d**) BS with (**a**,**b**) FOP and (**c**,**d**) HOP masks.

The success probabilities for different (L,δ) combinations are shown in Figure 11. With FOPs, a success probability of nearly 1 is achieved with (L≥5, δ≥5) and (L≥6, δ≥5) for the SGH and BS, respectively. On the other hand, with HOPs, the transition occurs with (L≥6, δ≥6) and (L≥6, δ≥8). In general, probe-based masks from FOPs require less oversampling than those from HOPs. In contrast, the required number of masks is similar. Both mask types provide a mask-dependent transition. The transition is not as good as for Gaussian masks but less mask-dependent than for categorical masks. In addition to the EES, Figure 12 shows the success probabilities based on the SMCs and the complex metric from (28). In contrast to the EES, a successful recovery is characterized by $\epsilon^{SMC}\leq -30\;\mathrm{dB}$. With this success-level definition, very similar probabilities for all (L,δ) combinations and transitions are achieved as with the EES metric.

**Figure 13:**
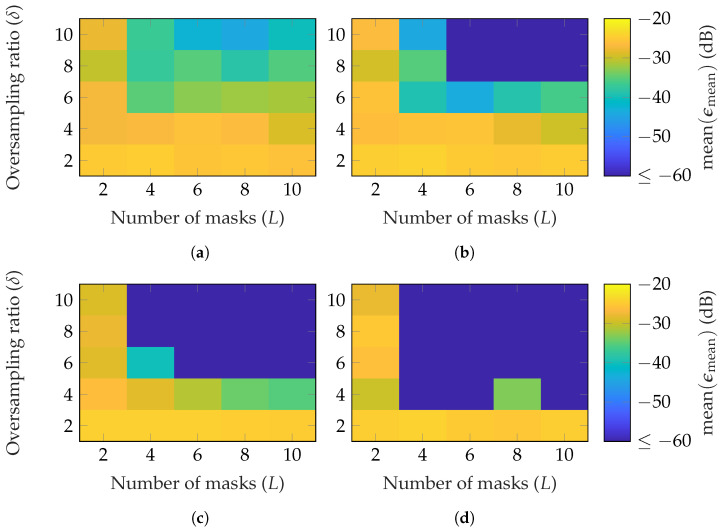
FF reconstruction error of the BS with random HOP masks with different mode truncation: (**a**) $N_{\mathrm{probe}}=7$, (**b**) $N_{\mathrm{probe}}=15$, (**c**) $N_{\mathrm{probe}}=22$, and (**d**) $N_{\mathrm{probe}}=38$.

Next, an important parameter to consider when generating FOPs or HOPs is their mode truncation. In Figure 13, the reconstruction performance with random HOP masks with different mode truncations is depicted. It can be seen that, as $N_{\mathrm{probe}}$ increases, the necessary number of masks and oversamplings required for a good reconstruction decreases. Therefore, mode truncation should be taken into account when creating probe-based masks.

**Figure 14:**
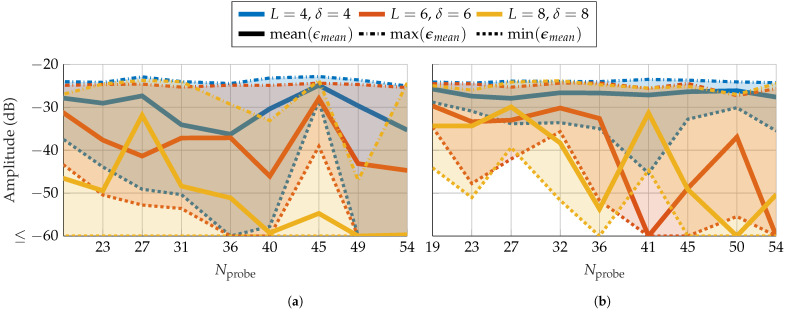
FF reconstruction error of (**a**) SGH and (**b**) BS with probe-based mask from patch arrays with different number of elements and thus mode truncation.

In the remainder of this section, a more practical approach of probe-based masks created from patch arrays is investigated. The same measurement radii as previously selected are used. The first step is to determine the number of elements the patch arrays should have. The array size is directly linked to its mode truncation number $N_{probe}$ and thus to the probe mode truncation $\nu_{\max}$ in (1). In Figure 14, similar to Figure 8, only three parameter combinations were simulated. This time, the array size is varied from 4 to 20 elements in steps of 2. The *x*-axis shows the respective mode truncation. The assignment between the number of array elements and the mode truncation is depicted in Table 2. For the SGH, the parameter combination (L=4, δ=4) shows, on average, a bad reconstruction over all patch arrays. (L=6, δ=6) as well as (L=8, δ=8) demonstrate a slightly decreasing error with increasing number of modes. However, for $N_{\mathrm{probe}}=27$ and especially $N_{\mathrm{probe}}=45$, the reconstruction error increases in contrast to the decreasing trend. Patch arrays with 14×14 ($N_{\mathrm{probe}}=40$), 18×18 ($N_{\mathrm{probe}}=49$) or more elements are best suited. For the BS, (L=4, δ=4) shows, on average, similar to the SGH, a bad reconstruction over all patch arrays. The error for (L=6, δ=6) and (L=8, δ=8) initially remains constant and later drops below −50 dB after $N_{\mathrm{probe}}=36$ and $N_{\mathrm{probe}}=41$, respectively. However, similar to the SGH, the error increases drastically for $N_{\mathrm{probe}}=41$ and $N_{\mathrm{probe}}=\left[45,50\right]$ after decreasing again. Thus, patch arrays with 12×12 ($N_{\mathrm{probe}}=36$) and 16×16 ($N_{\mathrm{probe}}=45$) elements are the best appropriate. The reason behind this non-monotonic behavior is an open concern and should be investigated in future works. Finally, it should be noted that the mode truncation is computed based on the physical size of the array and its frequency. The computation includes a constant whose value is chosen based on experimental data and satisfies all kinds of AUTs. However, for many antennas, the relevant modes do not range up to this truncation number; therefore, the effective mode truncation is lower. For example, for a 12×12 patch array, the mode truncation is set to $N_{\mathrm{probe}}=36$, but the effective one is a couple of modes lower.

**Figure 15:**
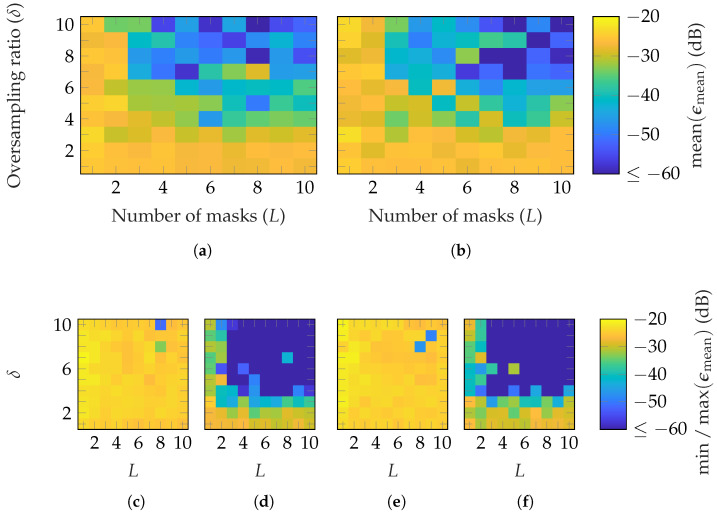
FF reconstruction error of SGH with probe-based masks from patch arrays with (**a**,**c**,**d**) 14×14 and (**b**,**e**,**f**) 18×18 elements.

**Figure 16:**
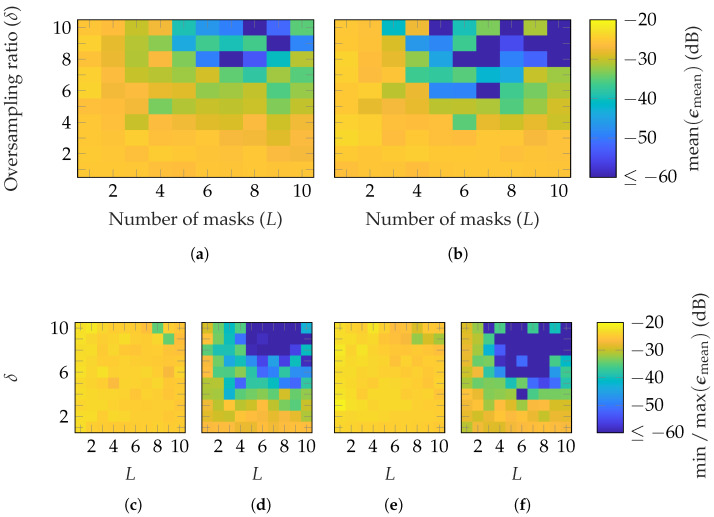
FF reconstruction error of BS with probe-based masks from patch arrays with (**a**,**c**,**d**) 12×12 and (**b**,**e**,**f**) 16×16 elements.

In Figure 15 and Figure 16, the reconstruction performance is shown for both AUTs and the previously selected patch array probes. For the SGH, on average, (L≥8, δ≥8) and (L≥7, δ≥7) are necessary to reach an $\epsilon_{mean}\leq -60\;\mathrm{dB}$ with 14×14 and 18×18 patch array probes, respectively. For the BS, (L≥7, δ≥8) and (L≥6, δ≥8) are required with 12×12 and 16×16 patch array probes, respectively. However, for both AUTs, a good reconstruction is not achieved for all (L,δ) combinations satisfying these bounds, as is the case for random probes. Moreover, in the worst case (see Figure 15c,e and Figure 16c,e), no good reconstruction is possible for the simulated parameter combinations. On the other hand, in the best case (see Figure 15d,f and Figure 16d,f), a good reconstruction is achieved with (L≥3, δ≥5), (L≥3, δ≥4), (L≥5, δ≥8) and (L≥5, δ≥6), respectively. A lower number of masks and oversampling is needed in the best case for the SGH than the BS for the chosen patch array probes.

**Figure 17:**
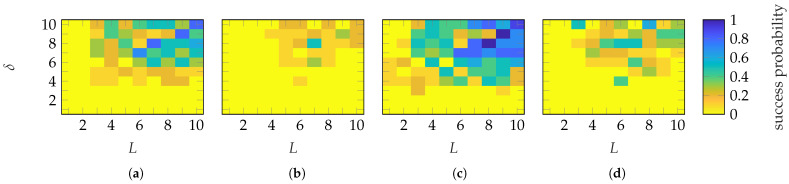
Success probability of the (**a**,**c**) SGH with 18×18 probe patch arrays and (**b**,**d**) BS with 16×16 probe patch arrays. Different success definitions are used: (**a**,**b**) with $\epsilon_{mean}\leq -60\;\mathrm{dB}$ and (**c**,**d**) with $\epsilon_{mean}\leq -40\;\mathrm{dB}$.

**Figure 18:**
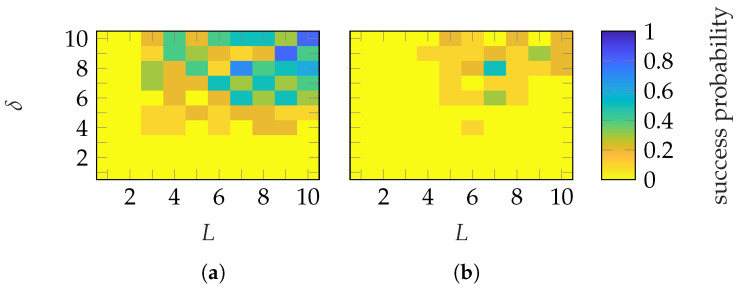
Success probability based on the SMCs metric ($\epsilon^{SMC}$) of the (**a**) SGH with 18×18 probe patch arrays and (**b**) BS with 16×16 probe patch arrays.

Figure 17 depicts the success probability of the two most promising patch array probes: 18×18 and 16×16 for the SGH and BS, respectively. The same success definition, as before, is used in Figure 17a,b. For the SGH, a success probability of 1 is only achieved for sparse parameter combinations. On the other hand, a maximum success probability of only 0.5 is reached for the BS. No abrupt or mask-dependent transition behavior is observed for either antenna. In Figure 17c,d the success definition is changed to $\epsilon_{mean}\leq -40\;\mathrm{dB}$. This error generally corresponds to well-reconstructed main beam and first side lobes, as will be shown in the next paragraph. Therefore, for the SGH, a success probability ≥0.6 is achieved for (L≥7, δ≥7) except for (L=7,8andδ=9) while for the BS, similarly as before, a maximum success probability of only 0.6 is achieved sparsely. Furthermore, here, it is not possible to define a sharp transition region in the joint dependency of (L,δ). Only for the SGH, 18×18 patch array probes and with $\epsilon_{mean}\leq -40\;\mathrm{dB}$, a mask-dependent transition can be observed, which is not as good as the one of random probes. Finally, similar to random probes, the success probabilities defined by $\epsilon^{SMC}\leq -30\;\mathrm{dB}$ and shown in Figure 18 are equivalent to the ones established by $\epsilon_{mean}\leq -60\;\mathrm{dB}$.

**Figure 19:**
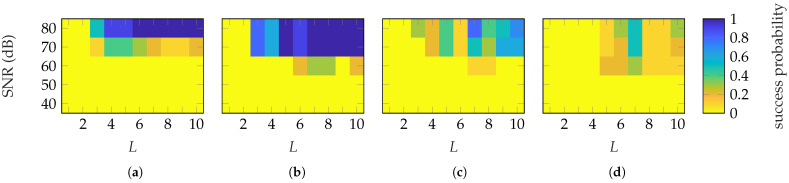
Success probability under the influence of noise of the SGH with masks based on (**a**) FOPs, (**b**) HOPs and (**c**) 18×18 patch arrays, and of the BS (**d**) with 16×16 patch arrays. The oversampling is fixed to δ=8.

To date, only ideal data have been considered. In Figure 19, the simulation results from reconstructions with Gaussian-distributed amplitude noise are shown. The amplitude noise is defined through a signal-to-noise ratio (SNR). It can be observed that an SNR of 80 dB is necessary to reach the same success rates as in the ideal case. However, there is an exception for the SGH and masks based on HOPs where an SNR of 70 dB is sufficient. Therefore, probe-based masks are prone to noise and require an SNR of 70,80 dB.

**Figure 20:**
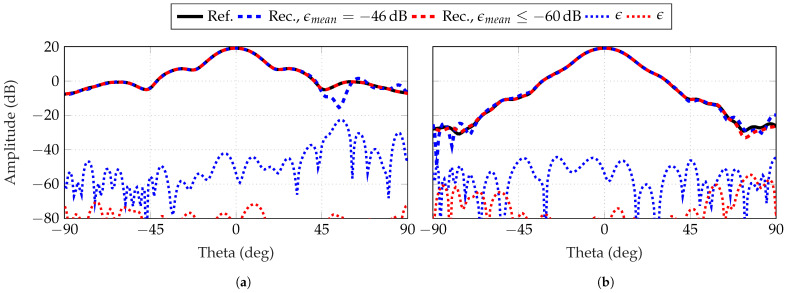
Reconstructed FF radiation pattern of SGH for (**a**) $\phi =0^{\circ }$ and (**b**) $\phi =90^{\circ }$ with probe-based masks from 18×18 patch arrays. Two simulations are chosen of (L=7, δ=7) with two different $\epsilon_{mean}$: below its $\mathrm{mean}(\epsilon_{mean})$ of −48 dB and one of a reconstruction with an error below −60 dB.

**Figure 21:**
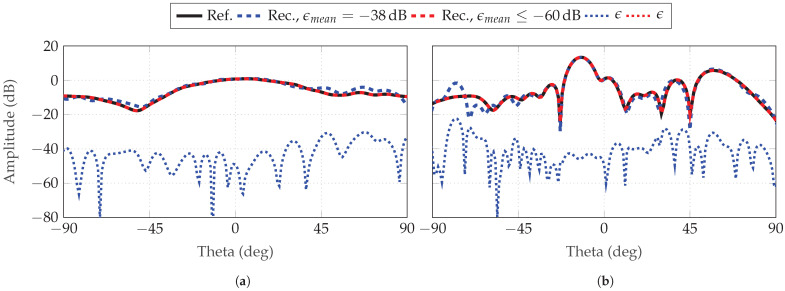
Reconstructed FF radiation pattern of BS for (**a**) $\phi =0^{\circ }$ and (**b**) $\phi =90^{\circ }$ with probe-based masks from 16×16 patch arrays. Two simulations are chosen of (L=8, δ=8) with two different $\epsilon_{mean}$: below its $\mathrm{mean}(\epsilon_{mean})$ of −49 dB and one of a reconstruction with an error below −60 dB.

Finally, Figure 20 and Figure 21 depict the reconstructed FF radiation patterns of the simulated AUTs, with the parameter combinations (L=7, δ=7) and (L=8, δ=8) for the SGH and the BS, respectively. Two simulations are selected for each combination. One with an $\epsilon_{mean}$ between $\min(\epsilon_{mean})$ and $\mathrm{mean}(\epsilon_{mean})$, and one with an $\epsilon_{mean}\geq -60\;\mathrm{dB}$. In all observed cases, the main lobe and side lobes are reconstructed even for the tilted beam of the BS. A max(ϵ) can be observed in Figure 20a and Figure 21b outside the main beam at $\theta =54^{\circ }$ and $\theta =-75^{\circ }$. As the reconstruction error decreases, from our observations, the main lobe is the region which is improved first and maximum errors tend to be located outside this angular region.

In conclusion, from the previous investigations, the design of probe-based patch arrays should be driven by the following guidelines:

- The distance between AUT and probe should be small to enhance the probe effect. In the conducted investigations, a measurement radius satisfying $r_{NF}\leq 0.2r_{FF}$ has been shown to be appropriate.
- The probe’s mode truncation should be large enough so that the probe effect is not negligible.
- The focus should be on the design of FOPs as a lower oversampling is necessary than for HOPs.

From a practical standpoint, the probe-based mask approach, as well as the mask approach in general, modify the measurement setup and procedure:

- The measurement time is mainly driven by the number of masks and oversampling. First, the oversampling ratio determines the number of sampling points and thus the sampling scheme of each mask measurement. Secondly, for each AUT, multiple mask measurements given by the number of masks have to be performed.
- The calibration of patch arrays is not necessary as long as the mode spectrum is known for probe correction. It is only a concern if a specific, pre-simulated mask, is required.
- For probe-based masks, the probe has to be changed at each mask measurement. This can be implemented via an automatic feed switcher or feed carousel. For patch arrays probes, phase shifters and attenuators could be used to change automatically the elements weights. However, this requires a more complex probe antenna.

## 5. Conclusions

A mask-based approach for phase recovery was presented and applied to SNF antenna measurements. Three types of masks were introduced and discussed. Simulations using a FF metric and an SMCs metric were employed to analyze their performance. Different parameters were investigated, with the two main ones being the number of masks and oversampling. The success rates over multiple simulations were analyzed and compared between the different masks. In addition, several AUTs with different directivities and beam tilts were used to demonstrate the robustness of the approach. The first type of mask is a random mask, demonstrating that this approach enhances phase reconstruction. Moreover, it has been shown that the type of random distribution affects the effectiveness of this approach, necessitating the use of more masks or a higher oversampling. The second type is a mask based on ϕ-rotations. This type has only been discussed, not investigated, as it is similar to oversampling, which has already been studied in the literature and shown to improve reconstruction to a limited extent. The third type is a probe-based mask. Random probes based on FOPs and HOPs have shown that this mask type can improve the phase reconstruction. Furthermore, FOPs require less oversampling, compared to HOPs, to achieve a good FF reconstruction. In a more practical approach, patch array probes with random element weighting have been used to generate multiple probes and thus masks. Depending on the array size, a good reconstruction can be achieved. However, more masks and a higher oversampling, as for random masks, are necessary, and the reconstruction is strongly dependent on the selected masks. The generated patch array probes were HOPs; therefore, future works could focus on the generation of FOP masks with enough diversity. Additionally, the reason behind the non-monotonic behavior of the reconstruction error with increasing array size should be further investigated. Further impairments, such as probe-pattern errors, radius miscalibration, polarization purity degradation, amplitude scale errors, and missing samples, should also be analyzed, in addition to the examined amplitude noise. Finally, selecting appropriate masks remains an open challenge that significantly impacts robustness, especially for probe-based masks.

## Figures and Tables

**Table 1:** AUTs and most important parameters.

AUT	Frequency (*f*)	Mode Truncation (*N*)	Number of Modes (*J*)	FF Radius at *f* ($r_{\mathrm{FF}}$)	Beam Tilt	Directivity
SGH	60 GHz	27	1566	0.3 m	0 deg	19 dBi
BS	2.4 GHz	31	2046	12 m	12 deg	16 dBi
mmVAST	19.76 GHz	160	51,840	69 m	0 deg	33 dBi

**Table 2:** Probe patch arrays and mode truncation number.

**Array elements one-dimensional ($N_{a}$)**	4	6	8	10	12	14	16	18	20
**Mode truncation ($N_{\mathrm{probe}}$) at 60 GHz**	18	23	27	31	36	40	45	49	54
**Mode truncation ($N_{\mathrm{probe}}$) at 2.4 GHz**	19	23	27	32	36	41	45	50	54

## Data Availability

The original contributions presented in this study are included in the article. Further inquiries can be directed to the corresponding author.

## Abbreviations

The following abbreviations are used in this manuscript:

SNF	spherical near-field
FF	far-field
NF	near-field
AUT	antenna under test
SMCs	spherical mode coefficients
PRCs	probe response constants
EES	equivalent error signal
SWE	spherical wave expansion
CDPs	coded diffraction patterns
FOP	first-order probe
HOP	high-order probe
SGH	standard gain horn
BS	basis station

## References

[1] Hansen J.E. (1988). Spherical Near-Field Antenna Measurements.

[2] Breinbjerg O. Spherical near-field antenna measurements—The most accurate antenna measurement technique. Proceedings of the 2016 IEEE International Symposium on Antennas and Propagation (APSURSI).

[3] Smith D., Leach M.P., Elsdon M., Foti S.J. (2007). Indirect Holographic Techniques for Determining Antenna Radiation Characteristics and Imaging Aperture Fields. IEEE Antennas Propag. Mag..

[4] Laviada Martínez J., Arboleya-Arboleya A., Álvarez-López Y., García-González C., Las-Heras F. (2014). Phaseless Antenna Diagnostics Based on Off-Axis Holography with Synthetic Reference Wave. IEEE Antennas Wirel. Propag. Lett..

[5] Arboleya A., Laviada J., Laurinaho J.A., Álvarez Y., Heras F.L., Räisänen A.V. (2016). Phaseless Characterization of Broadband Antennas. IEEE Trans. Antennas Propag..

[6] Candes E.J., Strohmer T., Voroninski V. (2013). PhaseLift: Exact and Stable Signal Recovery from Magnitude Measurements via Convex Programming. Commun. Pure Appl. Math..

[7] Candes E.J., Li X., Soltanolkotabi M. (2015). Phase Retrieval via Wirtinger Flow: Theory and Algorithms. IEEE Trans. Inf. Theory.

[8] Rodríguez Varela F., Fernández Álvarez J., Galocha Iragüen B., Sierra Castañer M., Breinbjerg O. (2021). Numerical and Experimental Investigation of Phaseless Spherical Near-Field Antenna Measurements. IEEE Trans. Antennas Propag..

[9] Fernández Álvarez J., Mattes M., Breinbjerg O. (2024). Phaseless Probe-Corrected Spherical Near-Field Antenna Measurements Using Two Scan Spheres. IEEE Trans. Antennas Propag..

[10] Fuchs B., Mattes M., Rondineau S., Le Coq L. (2020). Phaseless Near-Field Antenna Measurements From Two Surface Scans—Numerical and Experimental Investigations. IEEE Trans. Antennas Propag..

[11] Fernández Álvarez J., Bjørstorp J.M., Breinbjerg O. Spherical Phaseless Probe-Corrected Near-Field Measurements of the DTU-ESA VAST12 Reflector Antenna. Proceedings of the 40th Annual Meeting and Symposium of the Antenna Measurement Techniques Association.

[12] Rodríguez Varela F., Galocha Iragüen B., Sierra Castañer M., Fernández Álvarez J., Mattes M., Breinbjerg O. Combination of Spherical and Planar Scanning for Phaseless Near-Field Antenna Measurements. Proceedings of the 2019 Antenna Measurement Techniques Association Symposium (AMTA).

[13] Guth A., Culotta-López C., Maly J., Rauhut H., Heberling D. Polyhedral Sampling Structures for Phaseless Spherical Near-Field Antenna Measurements. Proceedings of the 2020 Antenna Measurement Techniques Association Symposium (AMTA).

[14] Paulus A., Knapp J., Eibert T.F. (2017). Phaseless Near-Field Far-Field Transformation Utilizing Combinations of Probe Signals. IEEE Trans. Antennas Propag..

[15] Fernández Álvarez J., Mattes M., Breinbjerg O. Phase Retrieval for Spherical Near-Field Measurements Using Two Antenna Positions. Proceedings of the 2021 Antenna Measurement Techniques Association Symposium (AMTA).

[16] Mézières N., Le Coq L., Fuchs B. (2022). Spherical Phaseless Antenna Measurements Experimental Validation of a Two-Antenna-Positions Procedure. IEEE Antennas Wirel. Propag. Lett..

[17] Kornprobst J., Paulus A., Knapp J., Eibert T.F. (2021). Phase Retrieval for Partially Coherent Observations. IEEE Trans. Signal Process..

[18] Paulus A., Knapp J., Kornprobst J., Eibert T.F. (2022). Reliable Linearized Phase Retrieval for Near-Field Antenna Measurements with Truncated Measurement Surfaces. IEEE Trans. Antennas Propag..

[19] Knapp J., Paulus A., Kornprobst J., Siart U., Eibert T.F. (2021). Multifrequency Phase Retrieval for Antenna Measurements. IEEE Trans. Antennas Propag..

[20] Candès E.J., Eldar Y.C., Strohmer T., Voroninski V. (2013). Phase Retrieval via Matrix Completion. SIAM J. Imaging Sci..

[21] Candès E.J., Li X., Soltanolkotabi M. (2015). Phase Retrieval from Coded Diffraction Patterns. Appl. Comput. Harmon. Anal..

[22] Guth A., Abdulsalaam S., Rauhut H., Heberling D. Numerical Investigations on Phase Recovery from Phaseless Spherical Near-Field Antenna Measurements with Random Masks. Proceedings of the 2024 Antenna Measurement Techniques Association Symposium (AMTA).

[23] Guth A.A., Abdulsalaam S., Rauhut H., Heberling D. Numerical Investigations on Phase Recovery from Phaseless Spherical Near-Field Antenna Measurements with Probe-Based Masks. Proceedings of the 2025 19th IEEE European Conference on Antennas and Propagation (EuCAP).

[24] Li H., Li S. (2021). Phase Retrieval from Fourier Measurements with Masks. Inverse Probl. Imaging.

[25] Gross D., Krahmer F., Kueng R. (2017). Improved recovery guarantees for phase retrieval from coded diffraction patterns. Appl. Comput. Harmon. Anal..

[26] Culotta-López C., Heberling D. Fast Spherical Near-Field Measurements on Arbitrary Surfaces by Application of Pointwise Probe Correction to Compressed Sampling Schemes. Proceedings of the 2019 Antenna Measurement Techniques Association Symposium (AMTA).

[27] Bangun A., Behboodi A., Mathar R. Signal Recovery from Phaseless Measurements of Spherical Harmonics Expansion. Proceedings of the 27th EUSIPCO 2019.

[28] Chandra R., Zhong Z., Hontz J., McCulloch V., Studer C., Goldstein T. PhasePack: A phase retrieval library. Proceedings of the 2017 51st Asilomar Conference on Signals, Systems, and Computers.

